# Home working at a public university due to the COVID-19 pandemic: challenges and opportunities

**DOI:** 10.47626/1679-4435-2022-837

**Published:** 2022-03-30

**Authors:** Aline Bicalho Matias, Isabela Saura Sartoreto Mallagoli

**Affiliations:** 1 Departamento de Saúde do Trabalhador, Universidade Federal de São Paulo, São Paulo, SP, Brazil.

**Keywords:** teleworking, universities, public sector, information access

## Abstract

**Introduction:**

The COVID-19 pandemic made it necessary to rapidly adapt ways of working, forcing adoption of home working, and public higher education institutions were no exception to this trend.

**Objectives:**

To analyze the initial phase of implementation of emergency teleworking at a public university, including its ramifications and repercussions for workers.

**Methods:**

This article constitutes a narrative account of the university’s experience with teleworking, evaluated by analysis of a report produced by the institution after a survey of the needs of workers assigned to emergency teleworking because of the health crisis. **Report of the experience:** The analysis identified both opportunities and challenges created by the experience, which involved 50% of the institution’s workers who were assigned to this modality of working. The most important opportunities were a perception of increased productivity compared to on-site working and improvements in quality of life and mental health. In counterpoint, barriers observed included worsening of mental health symptoms in the majority of workers who already had some type of mental disorder before the pandemic, work overload, difficulties with reconciling work routines with domestic activities, and a lack of the conditions and training needed to work from home.

**Conclusions:**

It should be emphasized that the various characteristics observed are related to the initial stages of adaptation to pandemic conditions and the new working routine. In counterpoint, some of the characteristics identified could offer important clues for continuation of teleworking and support for the university administration’s planning for the future.

## Introduction

Teleworking is nothing new in the corporate world and can be defined as a modality in which work is performed remotely using information and communication technologies.^1,2^ Although it has been described in the literature since the 1950s, teleworking is unusual in the context of public universities.^3,4^

As the COVID-19 pandemic arrived in Brazil, in March of 2020, the public health measures adopted to contain dissemination of the virus imposed a new and unexpected scenario of physical distancing, creating a need for universities to adapt their working methods.^5,6^ They were forced to take a rapid decision to implement teleworking, without the necessary planning and training.^7,8^

Aware that this urgent situation was far from ideal, university managers rapidly established standards to ensure teleworking was feasible. In contrast to what generally occurs in private firms, at public institutions, including universities, the costs of implementing the physical and technological infrastructure needed are borne by the workers, who also pay the electricity and internet bills, for example.^3^ These factors exacerbate the stressful situations already being experienced because of the newly-arrived pandemic and fear of contagion.^8^

The objective of this narrative account of the experience was to describe the analysis of an initial report on implementation of emergency teleworking by a public university in the Brazilian state of São Paulo during the COVID-19 pandemic, analyzing its ramifications and repercussions for workers, for their health, and for the institution.

## Methods

This article constitutes a narrative account of the experience of one university’s initiative to implement teleworking during the COVID-19 pandemic with the objective of mitigating dissemination of the virus and protecting the physical health of its workers. The experience was investigated by analyzing a report produced by the institution’s Occupational Health Department, which conducted a survey of the needs of workers assigned to emergency teleworking because of the public health crisis.

The survey was conducted during July of 2020, 3 months after the university had initiated emergency remote working. It was conducted by sending an on-line questionnaire to workers assigned to teleworking at that point, when 83% of teaching staff and 35% of educational technical-administrative staff were working from home. All of the information obtained, including data relating to health, constitute respondents’ self-reported perceptions.

## Narrative account of the experience

Analysis of the survey report identified difficulties and challenges that the university needed to address to enable teleworking to be maintained during the pandemic and for any future remote working projects. It also identified opportunities and benefits offered by this working modality. It should be pointed out that 50% of the institution’s employees were assigned to teleworking at the time of the survey, equating to a population of 2,778 workers. A total of 1,560 participants responded to the survey, 875 educational technical-administrative staff and 685 teaching staff.

Opportunities and benefits of the experience included protection from the virus and a perception of increased productivity compared with on-site working, especially with regard to meetings, with time savings because there was no need to travel between home and the workplace. In the individual sphere, there were many reports of improvements in quality of life, in nutrition, self-care, and mental health, in addition to emotional relief from workplace tensions. A study conducted with 906 workers in Paraná (another state in Brazil), the majority of whom were public sector employees, also underscored this perception of improved quality of life when working from home, compared to on-site working.^9^

In counterpoint, there were also many difficult experiences for the workers. They highlighted reductions in family income, difficulties with setting limits between work and leisure activities, difficulties reconciling work routines with domestic activities, low motivation to work, increased irritability, difficulties with concentration, and lack of face-to-face contact with work teams (Table 1). A predictive study about the impacts of Brazil’s labor law reform warned of the risks of sickness caused by stress and disruption of the private lives of workers working from home.^10^ Other studies have also highlighted these difficulties, in particular the superimposition of work over other activities and the difficulty of reconciling it with those activities, technological difficulties, the financial costs of teleworking, and the lack of contact with work colleagues.^2,9,11^

**Table 1:** Characteristics found by surveying the requirements of public sector employees at a public university assigned to emergency teleworking because of the COVID-19 pandemic

Characteristic	Yes n (%)	No n (%)
Changes to family income	936 (60.0)	624 (40.0)
High-quality internet access at home	1,375 (88.0)	185 (12.0)
Access to institutional data	975 (62.5)	585 (37.5)
Adequate environment for teleworking	959 (61.5)	601 (38.5)
Difficulty setting limits between work and leisure	936 (60.0)	624 (40.0)
Difficulty reconciling work with domestic activities	1,373 (88.0)	187 (12.0)
Perception that quality of work has deteriorated	608 (39.0)	952 (61.0)
Difficulty dealing with work colleagues	608 (39.0)	952 (61.0)
Perception of making more mistakes	592 (38.0)	968 (62.0)
Low motivation to work	847 (52.3)	713 (45.7)
Irritation with work	951 (61.0)	609 (39.0)
Difficulty concentrating	1,030 (66.0)	530 (33.0)
Difficulty organizing routines	967 (62.0)	593 (38.0)
Lack of face-to-face contact with work team	1,108 (71.0)	452 (29.0)
Worse bodily pain (posture and ergonomics)	715 (45.8)	845 (54.2)
Self-reported worsening of mental symptoms*	112 (52.0)*	104 (48.0)*
Emergence of mental symptoms	167 (10.0)	1,393 (90.0)

Source: Adapted by the authors from the report containing the results of the survey of workers’ needs.

* 216 employees already had mental disorders before the pandemic. The percentage was calculated based on this subset of workers.

Still with regard to the limitations of this modality of working, there were reports of a lack of the necessary conditions and of training on how to work from home, compounded by a lack of adaptation of procedures to conducting activities remotely and concerns with the confidentiality of activities carried out in virtual environments. A study conducted in 29 countries of the European Union highlighted the limitations of tools as one of the major barriers to working remotely.^12^ There were also reports of demands for productivity and for “clocking in” in the same manner as when working on site in a pandemic-free scenario, of excessive workload, of accumulation of roles, and of bosses/colleagues having difficulty understanding the limitations and problems imposed by the situation.

In the personal sphere, there were constant reports of spending on office materials and on resources to make work feasible, of significant increase in energy costs and internet bills, and of a home environment with unsatisfactory conditions to adequately carry out their work, whether because of ergonomics, noise, lack of sufficient space, and/or the need to share workspace. It was also observed that the mental symptoms of the majority of workers who had some type of preexisting mental disorder before the pandemic and the move to teleworking had worsened and that mental symptoms emerged in 10% of the home workers (Table 1). There were also many reports of neglecting ongoing health treatments because of fear of contamination, increased perception of irritability, impatience, anxiety, fear, discouragement and lack of motivation, irregular sleep, and sensations of incapacity to work because of the pandemic situation. Many of these factors were also described in other studies.^8^ In common with our findings, a study in Thailand with workers in home office regimes identified musculoskeletal and mental health problems as important impacts of this work modality on workers’ health.^13^

Still with regard to the barriers observed, there were difficulties understanding the deductions from remuneration made because the employee was working from home and with regard to nonexistence of benefits for those assigned to teleworking.

In immediate reaction, the university administration produced a manual containing guidance for workers and management covering the rights and responsibilities of home workers and best practices to facilitate coexistence within this working modality.

## Conclusions

We emphasize that this experience with teleworking was the result of implementation of an emergency teleworking system in exceptional circumstances. Many of the characteristics highlighted in the survey are linked to the early phase of the pandemic in Brazil and to initial adaptations to this new scenario and new work routine. It is therefore necessary to conduct further investigations into which characteristics are the result of the phase of the pandemic and which are caused by teleworking. Regardless, many characteristics, such as concerns with financial costs and about the resources needed for teleworking, increased workload, absence of face-to-face contact with work teams/colleagues, and difficulties reconciling work with other activities, should provide a foundation for future planning.

In parallel with the survey, the Federal Government published Normative Instruction No. 65,^14^ setting out, criteria, and procedures for the implementation of management programs and adequacy of activities that can be conducted at a distance. In turn, with the aim of regulating teleworking in the context of the university, in October of 2020 the institution created a Commission for Studies of the Implementation of Teleworking. It is hoped that this prior experience with teleworking, albeit in an atypical situation, can provide a foundation and information of relevance to the commission to support development of procedures, criteria, and requirements for teleworking at the institution.

Finally, we emphasize the need for further investigation into the appearance and exacerbation of mental symptoms perceived by the workers. Although they do not constitute disorders, these data are indicative of suffering and should be taken into consideration when defining the university’s teleworking policy.
